# Lived experiences of women who developed uterine rupture following severe obstructed labor in Mulago hospital, Uganda

**DOI:** 10.1186/1742-4755-11-31

**Published:** 2014-04-22

**Authors:** Dan K Kaye, Othman Kakaire, Annettee Nakimuli, Michael O Osinde, Scovia N Mbalinda, Nelson Kakande

**Affiliations:** 1 Department of Obstetrics and Gynecology, School of Medicine, College of Health Sciences, Makerere University, P.O. Box 7072, Kampala, Uganda; 2 Department of Obstetrics and Gynecology, Jinja Regional Hospital, Jinja, Uganda; 3 Department of Nursing, School of Health Sciences, College of Health Sciences, Makerere University, P.O. Box 7072, Kampala, Uganda; 4 Clinical, Operations and Health Services Research Program, Joint Clinical Research Centre, P.O. Box 10005, Kampala, Uganda

## Abstract

**Background:**

Maternal mortality is a major public health challenge in Uganda. Whereas uterine rupture remains a major cause of maternal morbidity and mortality, there is limited research into what happens to women who survive such severe obstetric complications. Understanding their experiences might delineate strategies to support survivors.

**Methods:**

This qualitative study used a phenomenological approach to explore lived experiences of women who developed uterine rupture following obstructed labor. In-depth interviews initially conducted during their hospitalization were repeated 3–6 months after the childbirth event to explore their health and meanings they attached to the traumatic events and their outcomes. Data were analyzed using thematic analysis.

**Results:**

The resultant themes included barriers to access healthcare, multiple “losses” and enduring physical, psychosocial and economic consequences. Many women who develop uterine rupture fail to access critical care needed due to failure to recognise danger signs of obstructed labor, late decision making for accessing care, geographical barriers to health facilities, late or failure to diagnose obstructed labor at health facilities, and failure to promptly perform caesarean section. Secondly, the sequel of uterine rupture includes several losses (loss of lives, loss of fertility, loss of body image, poor quality of life and disrupted marital relationships). Thirdly, uterine rupture has grim economic consequences for the survivors (with financial loss and loss of income during and after the calamitous events).

**Conclusion:**

Uterine rupture is associated with poor quality of care due to factors that operate at personal, household, family, community and society levels, and results in dire physical, psychosocial and financial consequences for survivors. There is need to improve access to and provision of emergency obstetric care in order to prevent uterine rupture consequent to obstructed labor. There is also critical need to provide counselling and support to survivors to enable them cope with physical, social, psychological and economic consequences.

## Introduction

Uterine rupture is a major obstetric complication of labour that significantly contributes to maternal and perinatal mortality and morbidity [1-4]. It is associated with immediate complications, such as severe anaemia, shock and a ruptured bladder, and women who survive may experience long-term complications, such as vesicovaginal fistula, foot drop and a subsequent infertility following hysterectomy or tubal ligation [5-9]. In contrast to extensive research on incidence and risk factors for maternal mortality, relatively less attention has been given to research on quality of life of women who, through access to life-saving care, survive severe obstetric complications (near miss). Information on the extent of postpartum maternal morbidity is limited, particularly in developing countries where morbidity is prevalent. Even where available, morbidity data from hospital-based studies is hard to interpret in terms of quality of life of survivors. Yet maternal near miss morbidity has short and long-term physical, social, cognitive and psychological effects on the survivors [10-13] which need to be addressed in order to restore their health. Available data does not shed light on the experiences of affected individuals and the meanings they attach to them.

The underlying factors that cause maternal morbidity and mortality may be analyzed using the Three Delays Model [14]. This model identifies three phases of delay: delay in seeking care, delay in reaching health facilities for care and delay in receiving adequate care when reaching a health facility [14-17]. This model is based on the assumptions that knowledge of danger signs and preparedness for addressing the complications ensures that predictable elements of the three phases of delay can be anticipated, identified in time and addressed promptly as they arise [14-17]. Delays in seeking care may be caused by failure to recognize signs of complications, failure to perceive the severity of illness, cost considerations and previous negative experiences with the healthcare system [14-17]. Delays in reaching care may be created by long distances from a woman’s home to a health facility, poor condition of roads, and absent or unaffordability emergency transportation [14-17]. Delays in receiving care may result from negative attitudes of healthcare providers, shortages of supplies and basic equipment, absence of patient care guidelines, inadequate or unskilled personnel, and poor referral system [17]. The delays are often interactive or multiplicative [14]. The underlying factors for uterine rupture include delays in seeking appropriate care at the onset of labour, a poor or non-existent referral system, non-attendance of antenatal care, and delay to receive care for obstructed labor.

Patient-reported outcomes are increasingly becoming relevant and necessary as measures of quality of life. This approach echoes the increased interest in evaluation and interpretation of the lived experiences of individuals as a proxy indicator of the quality of life. There is thus need to explore the aftermath of life-threatening obstetric complications, focusing on the health and wellbeing consequences of these events. Uterine rupture remains a public health problem in low income countries, such as Uganda [9,18-21]. Our aim was to explore and describe the experiences of survivors of uterine rupture, specifically how this childbirth complication adversely affects the lives and livelihoods of affected women, from their own perspective.

## Methods

### *Study setting*

This project involved research on women who ‘nearly died’ of pregnancy-related complications, but somehow survived, hence called near-miss morbidity. The study was conducted in Mukono, Wakiso and Mpigi districts of Central Uganda, from June 1, 2013 to August 30, 2013.

### *Theoretical framework*

The theoretical framework used to explore the meanings attached by survivors to this experience was adapted from Souza et al. [22], which was developed from the definition of a maternal *near miss*[23,24]. Conceptually, maternal near misses represent a point on a continuum between extremes of good health and death, where mothers develop severe obstetric morbidity and somehow survive, either due to luck or the health care they receive. Such individuals may eventually recover, become temporarily or permanently disabled or die [22-24]. The World Health Organization has developed tools [24] which could be used to identify maternal near misses. These tools utilize a combination of clinical signs/symptoms, management practices or presence of organ dysfunction. The analysis was informed by the concept of health (a state of complete physical, social, psychological and spiritual well-being, and not merely the absence of disease or infirmity).

### *Study design*

A qualitative study design was chosen to investigate the lived experiences of women with history of uterine rupture during childbirth. This paper analyses findings these intermittent in-depth interviews with 16 women who survived a clinically defined ‘near-miss’. These interviews were conducted as part of a prospective longitudinal study, which was a post-doctoral research project of the first author (DKK) entitled: *Evaluation and surveillance of the impact of maternal and neonatal near-miss morbidity on the health of mothers and infants in Jinja and Mulago hospitals.* The goal of the project of this mixed-methods study is to assess preventable factors associated with maternal and neonatal near miss morbidity, from the perspective of patients and healthcare providers.

### *Data collection*

Purposive sampling was used to select 16 participants who had uterine rupture during childbirth. Potential research participants were recruited from Mulago hospital at the time of childbirth, at which time they were requested to participate in an ongoing study. Telephone contacts and residential addresses were obtained from them during their hospital stay. Using contact addresses and directions obtained from the participants during this initial contact, the participants were traced to their individual villages and communities, where a second in-depth interview was conducted at a venue chosen by the participant, 3–6 months after the initial interview. Unstructured interviews were employed in order to enable the participants to express their views freely and the meanings they attached to their experiences. To clarify the questions, an interview guide (Table 1) was used. There was some flexibility in the order of questions and in the prompts employed to enhance understanding of the unique experiences of the participants. With the permission of the participants, the interviews were audiotaped in the local dialect (Luganda) and later translated into English. A journal was kept for recording detailed field notes about the cultural and contextual incidents that were heard, seen, experienced, and thought about during the process of data collection, in order to better comprehend and interpret the content of the interviews.

**Table 1:** Interview guide on lived experiences of women with uterine rupture

**Number**	**Issue explored**	**Question**
1	Personal characteristics of interviewee	Could you tell me all about yourself?
2	History of pregnancy, childbirth and circumstances in which uterine rupture injury occurred	Could you tell me about the circumstance of your pregnancy, childbirth and what happened thereafter?
3	Effect of uterine rupture on health in totality (physical, psychological and social well being).	Please tell me about your everyday experiences with history of uterine rupture. How do you feel about your current health situation? Probe about physical symptoms, social relations, socio-economic situation, psychological/emotion well being.
4	Impact of uterine rupture on social relationships and livelihood	Please tell me all about your social situation since the childbirth (probe for marital relations and social network).
5	Experiences of living as a survivor of uterine rupture	Tell me your everyday experiences as a woman with this problem. What challenges do you face as a result of your condition, regarding work, relationship with spouse, friends and family?
6	Coping as a survivor of uterine rupture	How have you been able to cope with the condition? What health problems have you experienced since childbirth?

### *Data analysis*

Thematic analysis was employed. This entailed reading and rereading transcribed interviews to gain insight and deeper meaning in order to identify themes and categories. The analysis was done concurrently with data gathering, which helped to know what to ask in the next interview and to cross check information from each interview with subsequent participants. This process made it possible to recognize the saturation point at which no new information emerged from the data.

### *Ethical considerations*

Ethical approval to conduct the study was obtained from the Ethics and research committees of Mulago hospital (REC 310–2012), the School of Medicine, Makerere University College of Health Sciences (REC 2012–172) and Uganda National Council for Science and Technology. Permission to conduct the study was obtained from the Department of Obstetrics and Gynaecology, Makerere University. All participants gave written informed consent to be interviewed and to have follow-up assessment for a period of six months after the initial hospitalization. They were also provided with counselling and treatment during this period, which was a follow up of the initial evaluation during their hospitalization (the time during which they had developed uterine rupture).

## Results

Table 2 shows a summary of the information about the 16 participants. All the participants had at least primary level of formal education. All were married before the development of uterine rupture. However, at the time of the second interview only eleven seven were still married. All the participants were previously gainfully employed, but at the time of the interviews only three remained employed. Five of them had developed a urinary fistula.

**Table 2:** Socio-demographic and reproductive history and childbirth outcomes of the participants

**Participant**	**Age**	**Parity**	**Education level**	**Marital status**	**Employment status after childbirth**	**Infant outcome**	**Marital status 4 months after childbirth**	**Whether uterus (removed or repaired)**
NK	18	1	Primary	Married	Unemployed	Died	Married	Repaired
^Ω^MA	21	2	Primary	Married	Unemployed	Died	Married	Removed
JM	21	2	University	Married	Employed	αDied	Separated	Repaired
SL	22	3	Primary	Married	Unemployed	Died	Married	βRepaired
HM	24	2	Secondary	Married	Unemployed	Died	Separated	Removed
^Ω^JN	24	2	Primary	Married	Employed	Died	Separated	βRepaired
^Ω^SK	26	5	Primary	Married	Unemployed	αDied	Separated	Removed
SN	27	3	Secondary	Married	Unemployed	αDied	Separated	Removed
MN	28	7	Secondary	Married	Unemployed	Alive	Married	Repaired
EK	28	3	University	Married	Unemployed	Died	Married	Removed
^Ω^AN	31	4	Secondary	Married	Unemployed	Died	Separated	Removed
LO	32	3	Secondary	Married	Unemployed	αDied	Separated	Repaired
SB	34	4	Secondary	Married	Unemployed	Died	Married	Removed
AB	36	4	Secondary	Married	Employed	αDied	Separated	Repaired
^Ω^HS	35	5	Secondary	Married	Unemployed	Died	Separated	Removed
DN	38	7	Primary	Married	Unemployed	Alive	Married	βRepaired

*ΩDeveloped a urinary fistula;* α *Early neonatal death;* β *Tubal ligation was done.*

### Barriers to access healthcare

For most women, labor started at home, and they were in labor for 1 to 3 days before seeking medical intervention. However, for two women, the labor started while they were in hospital awaiting an elective caesarean section. The former were initially attended to by untrained traditional birth attendants (TBAs). All but two participants had eventually lost their babies. While for most women the baby was still born, for others the baby was alive but “exhausted” and died in the intensive care unit. Where the baby was still born or an early neonatal death, the mother never had a chance to see the baby, as exemplified by one participant:

“I had labor pains that started in the night and continued throughout the following day up to the evening of the second day. I was in severe pain and was very tired. Then I suddenly felt something give way and then noted the bleeding. This got my mother very worried, so she arranged to take me to hospital. On arrival, they told me my baby was “tired” and therefore, I needed an urgent operation. I was only told that the baby was dead at the time of delivery, after two days, after persistent begging that my baby should be brought. I never even got a chance to see the baby, as my mother-in-law is the one who took away and buried the baby at her home”.

Many women experienced delays at home, at the TBAs or at the lower health clinic from where they were later referred or from where they referred themselves. Even where there were formal referrals, there was poor documentation of the complication. Mothers arrived when it was too late, as reported by one participant:

“I stayed there for two days. The labor pains were strong but the baby could not come. I stayed there until my abdomen appeared to be divided into two. Then all of a sudden, the labor pains stopped, and that continuous pain started. It was then that my husband’s decided to take me to the hospital”.

A similar experience was reported by another participant.

“I was put away in a room to labor all alone. I was in there for 4 days, until after consulting a midwife from the local village clinic, a decision was made to take me to hospital. But then it was late in the afternoon and transport was not readily available”.

Various reasons were given for delays to access hospital care for delivery, including inaccessibility of hospitals due to the long distances, transportation difficulties, financial constraints, and delays to make decisions regarding referral for hospital delivery. Most of the women reported that they did not have much say when it came to deciding where to give birth, even if they would have liked to be sent to the hospital to deliver. Rather, spouses, mothers or mothers-in-law made the decisions. For instance, a participant expressed her frustration:

“The men make such decisions. If I go to hospital and incur any cost, I cannot pay. Also, as a woman, I have no right to take such a major decision. It is the men who take such decisions. When I was in labor, my husband was not around. So I waited for him to return, and that kept me in labor for 2 days. If I had the power, at that time I would have gone to hospital”.

Even when the women reached the facilities, they did not receive adequate or appropriate treatment in a timely manner. Most participants reported experiencing some delays: delays to receive attention when they reached the hospital, delay to make the appropriate decision on whether they could deliver normally, and delay to access theatre for surgical operations when a decision to operate had been made. There were also delays for one team of health providers to hand over to another. The delays were attributed to several factors. These include shortages of skilled staff, failure to evaluate the information from the referral letters for women who had been referred with obstructed labor, inadequate clinical evaluation (very little history taken or incomplete examinations), perceived poor documentation, poor evaluation of clinical notes by teams that had been handed over to at times of staff changing shifts, missed diagnoses, insufficient communication among staff and inadequate monitoring. This delay to access care was a major problem, as one participant explained:

“As for hospital delivery, you take long to get attention or services. Some health care providers make wrong diagnoses or make wrong decisions. And when one group comes to replace the one that has been treating your condition, they change the treatment of the first group, without asking you any questions or examining you. One group tells you that you are for an operation and another group says there is nothing written. Sometimes, even when you get an operation, you are not informed of the reason why”.

Despite the prolonged labor, some participants attributed their fate condition to the interventions by healthcare providers at the hospital; for example, vacuum extraction, and caesarean section. In their understanding, it was the interventions that may have caused the uterine rupture and associated injuries. One woman who developed an obstetric fistula believed the problem should be blamed on healthcare providers:

“I have given birth three times but never experienced such a thing. I had delivered before, actually twice, with no problem. I developed this condition following my fourth delivery in the hospital. Remember I had two home deliveries without any problem. I was taken to hospital after 2 days in labor. The doctor pushed a tube inside me to bring out the urine but only blood came out. He then used some instruments to pull out the baby by force, without success. When this failed, another doctor said I needed an operation. I think the doctor (who made attempts to deliver me with their instruments) is responsible. He must have caused the injury to my uterus and urinary system”.

Some participants, however, attributed their problem to incompetence or negligence of the healthcare providers, and believed that their outcome could have been different if the healthcare providers were more caring. This sentiment is exemplified by one participant, who had been referred from a clinic with obstructed labor, but delayed to receive appropriate care:

“I was calling the doctors (healthcare providers) nurse, but no one was listening. I was telling them that where I was referred from, I was told my baby was exhausted and that I would not manage to deliver. I told them that the contractions were very strong, but all they told me was that I should wait. By chance, one woman who was attending to another mother came and looked at me and alerted some doctor whom she knew personally. When that doctor checked me, he rebuked the team that had been looking after me, and immediately ordered that they take me next for the operation. He actually worked on me himself. What upsets me is that other doctors (healthcare providers) were just by-passing me. By the time they took me to theatre, my uterus had ruptured. I was just fortunate that the baby was saved. However, that doctor told me that it will not be possible for me to have more children”.

Participants expressed concern about the financial cost of hospital delivery. The delivery itself did not have an official fee, but most women reported paying some money to healthcare providers for services. Three women reported that they had multiple operations, as they had to be referred to the surgical theatre due to complications. They also spent a lot of money on drugs. All the participants spent more than ten days in hospital, and for four patients, the duration of hospital stay was close to one month. This cost was on top of money for transport and feeding while at the hospital. Some participants reported that informal payments were rife, and were both solicited and unsolicited. One participant developed the uterine rupture while in hospital, and spent several days while waiting to have an elective caesarean section. She reported several such transactions:

“The hospital expenses are a problem. Though officially services are free, you have to pay some money to get an operation.....I had to wait for two weeks. No one asked me for money directly. My neighbours told me that is why I am not taken (for operation). Some reported that they were asked directly for something before they could be operated”.

### Experience of living with uterine rupture: multiple losses

Almost all the participants reported suffering some degree of physical complications associated with uterine rupture. These ranged from urinary symptoms (urinary incontinence from obstetric fistula, stress incontinence or minor urinary symptoms such as dysuria), dyspareunia, low abdominal pain, backache, reduced or scanty menses and urinary tract infection. Those with with obstetric fistulas reported often experiencing pain secondary to urinary tract infection. Some women reported what seemed like premature menopausal symptoms, such as vaginal dryness, severe backache, sudden episodes of sweating, heat intolerance and unexplained palpitations. The premature menopause is exemplified by one participant:

“The backache is unbearable. And all of a sudden you begin sweating, even at night.. My skin is dry. I don’t feel like I am myself. And my heart beats very fast, even when I have not done much activity. I am so dry down (in my private parts) as if I got sores down. Sometimes the area becomes so itchy that I am not able to bear it. Whenever this happens, I scratch there and that tends to worsen the itching. I often get fever, loss of appetite, and severe abdominal pain”.

Two participants experienced foot drop, which resulted from prolonged pressure on the nerves supplying the lower limbs. This followed obstructed labor which was complicated by uterine rupture and an obstetric fistula. One of the participants who experienced foot drop reported:

“I could not move my leg initially, so I was just lying down. I stayed at the hospital for some time and was discharged without even regaining use of my legs. I left the hospital with a urinary tube and a bag, after 1 week in hospital. Before I went to have the tube removed, the urine started coming from my private parts. Leaking continued after the tube was removed. ....I was told to go back after three months, but I have not been able to go. I walk with difficulty. I am not sure whether I will ever regain normal function”.

Participants spoke with emotion about the way other people, especially their loved ones, treated them. They appeared to suffer greatly due to stigma: As one participant put it,

“I feel lonely. Everyone says I have bad luck. My husband said he cannot suffer with my problem when he has not even benefited from a single child from me. My first child died three days after birth. I wish my baby had survived. Then at least that would console me”.

Participants also expressed the pain of losing a baby and of being childless. To worsen this pain was the realization that there was no hope of ever becoming a mother, as one participant reported:

“I am disturbed. Apart from the agony of losing my baby, they told me my uterus was affected. I have not seen my menses. The blood just remains inside me. It is now impossible for me to ever become a mother. I am no longer a woman. A woman should see her periods. It is men who do not get periods. Again I am not a man. If I had the baby, I would have my consolation. Sometimes I sit alone in the house and cry from morning to evening without anyone to console me”.

Similar sentiments and experiences were expressed by other participants. One participant who had two previous surgical operations during delivery had delivery by emergency caesarean section. Unfortunately, her baby died on the second day after birth. She had resumed her menses, but was informed there was no hope for her of ever having children, as her uterus had ruptured and her uterine tubes had to be ligated:

“I get my periods but I think they are heavier and last for much longer. I also get pain in my abdomen, which I did not have before. I am lucky my uterus was not removed. But I was told it will never be possible for me to have more children”.

However, another participant was not so lucky, as her uterus was removed. Subsequently, she had not resumed her menses. Unfortunately, she also described what appeared to be menoupasal symptoms. She had a hysterectomy for uterine rupture five months earlier complicated by a pelvic abscess, and described her situation as follows:

“I have not got my menses. My co-wife insults me. When drunk, she tells her friends that I am another man in my house. In fact, I feel frustrated and hurt. I was informed that there is no hope of ever seeing my menses or getting children, I am now neither a woman nor a man. I don’t know what I am. I am just there”.

For five women, uterine rupture was associated with an obstetric fistula. These participants described the stigma of urinary incontinence, which led to social isolation. This stigma often led to self-isolation in order to avoid embarrassment and humiliation.

“It has affected my private and public life. I cannot control my urine. I have to keep on padding myself all the time. I no longer share the bed with my husband. I also rarely move out of home. I avoid public places. Because when I am in public, I worry about the smell. .... and when I my clothes get wet, it is embarrassing. It is better to stay at home”.

### Disruption of marital relationships

The participants reported a number of challenges of included stigma and social isolation, worry, the emotional pain of stigmatization, marital disruption, and limited social support. The majority of the women indicated that they ceased to enjoy sex in their marriage. A number of participants were still living in their marital home, but, for most, their relationship with their spouse had ended. Participants reported experiencing neglect or abandonment when their husbands turned away from them to other wives:

“I have a lot of pain when I attempt to have sex. So it is a burden. When I complain of being sick, he won’t even ask how I am doing. I never wanted this to happen. I am not even to blame. Ever since the delivery, we have rarely been together as husband and wife. I am just living in his house. I consider myself as still married but I am not”.

#### Enduring psychosocial, socio-economic and physical consequences

The participants also reported loss of social support, and that the only reliable family members were their children and siblings. The support received was mainly provision for basic needs, such as food and money. Some participants mentioned receiving money for treatment, or being taken to the hospital. While such support helped, it usually did not suffice to meet their needs. Mainly because of the severe obstetric condition, the women lost the jobs that had been their means of livelihood. At the same time, they incurred financial strain due to their condition. All of this led to deepening poverty and having to struggle to survive. Mostly self-employed in petty trading before the childbirth, the participants reported losing their business. Having lost their sources of livelihood, participants had insufficient income to meet their basic needs. Meanwhile, they had to spend more to keep clean and to pay the cost of treatment for their condition. Many were unsure yet of what the future would be, and were resigned to their fate, as exemplified by one participant:

“I don’t know what will happen next. Each day seems to bring new problems. As for my marriage, I hope for the best, but have accepted this as my destiny. I have to accept whatever comes. My stay will depend on them (my partner or his relatives). If they say I have to leave because all the children are dying, I will go back at my parents home. I will forget about marriage”.

## Discussion

The study presents the women’s experience of uterine rupture from the circumstances during which the complication occurred to how it eventually affected their lives in its entirety: physical disability, disruption of marital relationships and loss of opportunities to participate in income generating activities. The barriers to accessing health care provide both the background and context of the ‘loss’ of health and livelihood whereby women’s experiences are influenced by negative experiences of the traumatic childbirth. This study reveals that lived experiences of women with severe birth injuries such as uterine rupture are compounded by the negative experiences of the traumatic childbirth. The childbirth experiences are characterised by poor quality of care due to delay to receive prompt care and negative attitudes of staff. Similar findings have been reported on experiences of women with maternal near miss, which are described as a maternal syndrome [23] in which the experiences of a critical illness and those of the care overlap. Similar findings have been reported in women who developed obstetric fistula, another complication of obstructed labor, in Ghana [25] and Tanzania [26].

The study findings shed light on the risks associated with pregnancy and childbirth. They assess the performance of health systems in terms of access to health care, the quality of care provided and the quality of life of survivors. The participants described a continuum of ‘losses’: financial losses due to hospitalization costs, severe injuries (such as hysterectomy), persistent morbidity, loss of marital relationships, death of the newborns, loss of income and stigma (associated with loss of self-esteem and prestige), all of which contributed to their suffering. This finding is in agreement with previous research [11-13,22,23] that physical injury, disability, social isolation and psychological distress overlap in the aftermath of maternal near miss morbidity.

The ‘maternal near miss syndrome’ [23] is characterised by poor quality of life due to both illness and poor quality of care. The burden of maternal ill-health extends beyond physical complications and includes different short- and long-term morbid conditions that can result from acute obstetric complications or their sequel. The postpartum morbid consequences of traumatic childbirth include postpartum infection, anaemia, perineal tears, urinary tract infection, depression; incontinence, fistula, pelvic inflammatory disease, genital prolapse, hypertension, haemorrhoids, nerve damage, pituitary failure, anaemia, and infertility and post-traumatic stress disorder [27-36]. In our study, some women who sustained uterine rupture presented with the “obstructed labor injury complex”, which includes fetal death, fistula formation, secondary infertility, foot drop, stigmatization, isolation and loss of support, divorce or separation, worsening poverty, worsening malnutrition, and suffering, illness, and premature death.

The physical, social, psychosocial and socioeconomic problems that we found, including stigma and social isolation, a disrupted marital relationship, loss of livelihood, and worsening poverty, were also identified in other studies [37-41]. Many of the maternal morbidities and disabilities may arise during delivery, might appear any time within the subsequent six months, or could become chronic with worsening disability if not appropriately addressed [22,23,37,38]. The defining characteristics of ‘loss’ in ‘maternal near miss syndrome’ is psychological, social and biomedical, involving physical disruption, and loss of body image, social relations, livelihood and familiar patterns of life [39,40].

The study findings reveal how severe maternal morbidity is a gender issue that needs urgent attention. Firstly, maternal morbidity incurs economic costs which may be formal or informal [41]. Secondly, in addition to physical and social problems, women who were once economically productive and socially respected in their communities lose their income, productivity, dignity, image, identity, status, self-esteem and future capability as mothers [37-39]. Therefore, the aftermath of severe obstetric morbidity is characterized by a medical, social and financial crisis for many mothers [39]. Women’s accounts of their after a near-miss event show that such events do not only affect health in its entirety [11] but raise gender concerns that need to be addressed.

The limitations of the study are that it involved small numbers of affected women, and the period of follow up was only six months. However, the interviews were conducted on more than one occasion, and the second interviews were conducted away from a helth facility setting. The findings suggest that gender issues (women's status, women empowerment, employment, and decision-making) affect their reproductive health, particularly access to and use of services during pregnancy and childbirth. Secondly, gender concerns related to outcomes of childbirth have grave implications for the long term wellbeing and quality of life of mothers. Lastly, severe maternal illness is costly for families because of high direct health costs, loss of income, loss of economic productivity, disturbed family relationships, and social stresses. To increase likelihood of survival and improve quality of life, interventions should address access to emergency obstetric care and provide the continuum of social support to mitigate the gender and other non-obstetric causes of morbidity among survivors.

## Conclusion

Survivors of obstetric near miss events such as uterine rupture have subsequent physical, social socio-economic and psychological complications that diminish their quality of life. Disruption occurs in all domains (physical, social, sexual and psychological) and survivors’ lives are often severely affected by the traumatic events of childbirth. The findings underscore the need to routinely inquire about and address quality of life and gender issues of survivors of maternal near miss obstetric events. The results highlight the urgent need to identify at-risk populations for postpartum morbidity, the markers of persistent severe morbidity and appropriate interventions for support of the survivors. There urgent need to address third delay at the health facilities since facility level preparedness and ability to respond to obstetric emergencies is critical for the survival of women and reduction of obstetric morbidity and subsequent disability.

## Competing interests

The authors declare that they have no competing interests.

## Authors’ contributions

DKK conceptualized the study as part of his post-doctoral research project. OK, MOO, and NK advised on the design. DKK collected the data, led the analysis, and wrote the text of the paper. All the co-authors gave advice on the data analysis, presentation of the results. All co-authors reviewed and edited the text and approved the final manuscript.

## References

[B1] Hogan MC Foreman KJ Naghavi M Ahn SY Wang M Makela SM Lopez AD Lozano R Murray CJ Maternal mortality for 181 countries, 1980–2008: a systematic analysis of progress towards millennium development goal 5 Lancet 2010 375 9726 1609 1623 10.1016/S0140-6736(10)60518-1 20382417

[B2] World Health Organization Trends in maternal mortality 1990 to 2008, Estimates developed by WHO, UNICEF, UNFPA and the world bank 2010 Geneva: WHO Press

[B3] Khan KSW WHO analysis of causes of maternal death: a systematic review Lancet 2006 367 1066 1074 10.1016/S0140-6736(06)68397-9 16581405

[B4] Kaye DK Kakaire O Osinde MO Systematic review of the magnitude and case fatality ratio for severe maternal morbidity in sub-Saharan Africa between 1995 and 2010 BMC Pregnancy Childbirth 2011 11 65 10.1186/1471-2393-11-65 21955698 PMC3203082

[B5] Ezechi OC Mabayoje P Obiesie LO Ruptured uterus in South Western Nigeria: a reappraisal Singapore Med J 2004 45 113 116 15029412

[B6] Gessessew A Melese MM Ruptured uterus – eight year retrospective analysis of causes and management outcome in Adigrat Hospital, Tigray Region, Ethiopia Ethiopian J Health Dev 2002 16 241 245

[B7] Adamu RM Obed SA Ruptured uterus: a seven-year review of cases from Accra, Ghana J Obstet Gynaecol Can 2003 25 225 230 12610675 10.1016/s1701-2163(16)30110-4

[B8] Akaba GO Onafowokan O Offiong RA Omonua K Ekele BA Uterine rupture: trends and feto-maternal outcome in a Nigerian teaching hospital Niger J Med 2013 22 4 304 308 24283089

[B9] Wandabwa J Doyle P Todd J Kiondo P Wandabwa MA Aziga F Risk factors for ruptured uterus in Mulago Hospital Kampala, Uganda East Afr Med J 2008 85 56 63 18557248 10.4314/eamj.v85i2.9607

[B10] Elmir R Schmied V Wilkes L Jackson D Separation, failure and temporary relinquishment: women’s experiences of early mothering in the context of emergency hysterectomy J Clin Nurs 2012 21 7–8 1119 1127 22176681 10.1111/j.1365-2702.2011.03913.x

[B11] Pacagnella RC Cecatti JG Camargo RP Silveira C Zanardi DT Souza JP Parpinelli MA Haddad SM Rationale for a long-term evaluation of the consequences of potentially life-threatening maternal conditions and maternal “near-miss” incidents using a multidimensional approach J Obstet Gynaecol Can 2010 32 8 730 738 21050503 10.1016/s1701-2163(16)34612-6

[B12] de la Cruz CZ Coulter ML O’Rourke K Amina Alio P Daley EM Mahan CS Women’s experiences, emotional responses, and perceptions of care after emergency peripartum hysterectomy: a qualitative survey of women from 6 months to 3 years postpartum Birth 2013 40 4 256 263 10.1111/birt.12070 24344706

[B13] Elmir R Schmied V Jackson D Wilkes L Between life and death: women’s experiences of coming close to death, and surviving a severe postpartum haemorrhage and emergency hysterectomy Midwifery 2012 28 2 228 235 10.1016/j.midw.2010.11.008 21251734

[B14] Thaddeus S Maine D Too far to walk: maternal mortality in context Soc Sci Med 1994 38 1091 1110 10.1016/0277-9536(94)90226-7 8042057

[B15] Pacagnella RC Cecatti JG Osis MJ Souza JP The role of delays in severe maternal morbidity and mortality: expanding the conceptual framework Reprod Health Matters 2012 20 39 155 163 10.1016/S0968-8080(12)39601-8 22789093

[B16] Combs Thorsen V Sundby J Malata A Piecing together the maternal death puzzle through narratives: the three delays model revisited PLoS One 2012 7 12 e52090 10.1371/journal.pone.0052090 23284882 PMC3526530

[B17] Knight HE Self A Kennedy SH Why are women dying when they reach hospitalon time? A systematic review of the ‘third delay’ PLoS One 2013 21 8(5) e63846 23704943 10.1371/journal.pone.0063846 PMC3660500

[B18] Kaye DK Kakaire O Osinde MO Maternal morbidity and near-miss mortality among women referred for emergency obstetric care in rural Uganda Int J Gynaecol Obstet 2011 114 1 84 85 10.1016/j.ijgo.2011.01.026 21570683

[B19] Kaye D Mirembe F Aziga F Namulema B Maternal mortality and associated near-misses among emergency intrapartum obstetric referrals in Mulago Hospital, Kampala, Uganda East Afr Med J 2003 80 3 144 149 12762430 10.4314/eamj.v80i3.8684

[B20] Mukasa PK Kabakyenga J Senkungu JK Ngonzi J Kyalimpa M Roosmalen VJ Uterine rupture in a teaching hospital in Mbarara, western Uganda, unmatched case- control study Reprod Health 2013 10 1 29 10.1186/1742-4755-10-29 23718798 PMC3668214

[B21] Kadowa I Ruptured uterus in rural Uganda: prevalence, predisposing factors and outcomes Singapore Med J 2010 51 1 35 38 20200773

[B22] Wilson RE Salihu HM The paradox of obstetric “near misses”: converting maternal mortality into morbidity Int J Fertil Womens Med 2007 52 2–3 121 127 18320871

[B23] Souza JP Cocotte JG Parpinelli MA Krupa F Osis MJ An emerging “maternal near-miss syndrome”: narratives of women who almost died during pregnancy and childbirth Birth 2009 36 2 149 158 10.1111/j.1523-536X.2009.00313.x 19489809

[B24] Say L Souza JP Pattinson RC Maternal near miss – towards a standard tool for monitoring quality of maternal health care Best Prac Res Clin Obstet Gynecol 2009 23 287 296 10.1016/j.bpobgyn.2009.01.007 19303368

[B25] Mwini-Nyaledzigbor PP Agana AA Pilkington FB Lived experiences of Ghanaian women with obstetric fistula Health Care Women Int 2013 34 6 440 460 10.1080/07399332.2012.755981 23641897

[B26] Mselle LT Moland KM Mvungi A Evjen-Olsen B Kohi TW Why give birth in health facility? Users’ and providers’ accounts of poor quality of birth care in Tanzania BMC Health Serv Res 2013 13 174 10.1186/1472-6963-13-174 23663299 PMC3654954

[B27] Filippi V Ganaba R Baggaley RF Marshall T Storeng KT Sombié I Ouattara F Ouedraogo T Akoum M Meda N Health of women after severe obstetric complications in Burkina Faso: a longitudinal study Lancet 2007 370 1329 1337 10.1016/S0140-6736(07)61574-8 17933647

[B28] Ronsmans C Achadi E Cohen S Zazari A Women’s recall of obstetric complications in South Kalimantan, Indonesia Stud Fam Plann 1997 28 203 214 10.2307/2137888 9322336

[B29] Prual A Huguet D Garbin O Rabe G Severe obstetric morbidity of the third trimester, delivery and early puerperium in Niamey (Niger) Afr J Reprod Health 1998 2 10 19 10214424

[B30] Chama CM El-Nafaty AU Idrisa A Caesarean morbidity and mortality at Maiduguri, Nigeria J Obstet Gynaecol 2000 20 45 48 10.1080/01443610063453 15512465

[B31] Waterstone M Wolfe C Hooper R Bewley S Postnatal morbidity after childbirth and severe obstetric morbidity BJOG 2003 110 128 133 12618155

[B32] Filippi V Goufodji S Sismanidis C Kanhonou L Fottrell E Ronsmans C Alihonou E Patel V Effects of severe obstetric complications on women’s health and infant mortality in Benin Trop Med Int Health 2010 15 6 733 742 10.1111/j.1365-3156.2010.02534.x 20406426 PMC3492915

[B33] Gülmezoglu AM Say L Betrán AP Villar J Piaggio G WHO systematic review of maternal mortality and morbidity: methodological issues and challenges BMC Med Res Methodol 2004 4 16 10.1186/1471-2288-4-16 15236664 PMC481067

[B34] AbouZahr C Global burden of maternal death and disability Br Med Bull 2003 67 1 11 10.1093/bmb/ldg015 14711750

[B35] Vallely L Ahmed Y Murray SF Postpartum maternal morbidity requiring hospital admission in Lusaka, Zambia—a descriptive study BMC Pregnancy Childbirth 2005 5 1 10.1186/1471-2393-5-1 15686592 PMC549039

[B36] Bang RA Bang AT Reddy MH Deshmukh MD Baitule SB Filippi V Maternal morbidity during labour and the puerperium in rural homes and the need for medical attention: a prospective observational study in Gadchiroli, India BJOG 2004 111 231 238 10.1111/j.1471-0528.2004.00063.x 14961884

[B37] Storeng KT Murray SF Akoum MS Ouattara F Filippi V Beyond body counts: a qualitative study of lives and loss in Burkina Faso after ‘near-miss’ obstetric complications Soc Sci Med 2010 71 10 1749 1756 10.1016/j.socscimed.2010.03.056 20541307

[B38] Storeng KT Drabo S Ganaba R Sundby J Calvert C Filippi V Mortality after near-miss obstetric complications in Burkina Faso: medical, social and health-care factors Bull World Health Organ 2012 90 6 418 425 22690031 10.2471/BLT.11.094011 PMC3370364

[B39] Storeng KT Akoum MS Murray SF ‘This year I will not put her to work’: the production/reproduction nexus in Burkina Faso Anthropol Med 2013 20 1 85 97 10.1080/13648470.2012.692360 22897630 PMC3962072

[B40] Polachek IS Harari LH Baum M Strous RD Postpartum post-traumatic stress disorder symptoms: the uninvited birth companion Isr Med Assoc J 2012 14 6 347 353 22891394

[B41] Riewpaiboon W Chuengsatiansup K Gilson L Tangcharoensathien V Private obstetric practice in a public hospital: mythical trust in obstetric care Soc Sci Med 2005 61 7 1408 1417 10.1016/j.socscimed.2004.11.075 16005776

